# Fractalkine (Chemokine CX3CL1) Signaling During Placentation and Placental Function

**DOI:** 10.3390/ijms27031172

**Published:** 2026-01-23

**Authors:** Dariusz Szukiewicz

**Affiliations:** Department of Biophysics, Physiology & Pathophysiology, Faculty of Health Sciences, Medical University of Warsaw, 02-004 Warsaw, Poland; dariusz.szukiewicz@wum.edu.pl

**Keywords:** fractalkine, chemokine CX3CL1, fractalkine signaling, fractalkine receptor, CX3CR1, placenta, placental function, pregnancy, pregnancy complications

## Abstract

Precise postimplantation regulation of placental development with trophoblast invasion of uterine spiral arteries and the generation of low-resistance circulation within the utero-fetal unit are crucial for the further development of pregnancy. Cytokines, including chemokines, are crucial for ensuring placental function throughout pregnancy. The CX3CL1 chemokine (fractalkine), occurring in its membrane-bound form and as a soluble chemokine (sCX3CL1), acts on its sole receptor, namely, CX3CR1, creating a signaling axis that orchestrates the balance of cellular interactions, immune responses, and tissue remodeling needed at every stage of a healthy pregnancy. CX3CL1/CX3CR1 signaling is characterized by the activation of several downstream signaling cascades that interact with numerous pathways, coordinate with other receptors and modulate the expression of relevant genes. This review presents the current state of knowledge regarding the role of CX3CL1 and its interaction with CX3CR1 in establishing placental homeostasis during placentation, and it discusses the contribution of disturbances in this interaction to placental dysfunction. These disturbances are part of the pathomechanisms of specific pregnancy complications, including preeclampsia (PE) and diabetes. The potential to target the CX3CL1/CX3CR1 axis via therapeutic intervention at the level of the placenta in PE- and diabetes-complicated pregnancy is the subject of ongoing research.

## 1. Introduction

The placenta is a transient, multifunctional organ unique to each pregnancy, acting as the lungs, liver, kidneys, and endocrine glands for the fetus until fetal organs develop and are functional, which occurs after birth in the case of the lungs [1]. The placenta forms a unique union of both maternal and fetal tissues, managing complex exchanges between the mother and the child and influencing the physiology of the mother [2].

Cytokines, including chemokines, are crucial for ensuring placental function throughout pregnancy. Cytokine signaling is involved in implantation, placental development, angiogenesis, immune regulation, and childbirth [3,4,5]. Imbalances in cytokine signaling underlie pregnancy complications, such as recurrent miscarriage, preeclampsia (PE), fetal growth retardation (FGR) and preterm birth [6,7,8,9,10].

In 1997, two independent research teams, led by Bazan and Pan, described an unusual cytokine, CX3CL1 (fractalkine or neurotactin), that exists in two forms—soluble and membrane-bound [11,12]. The soluble form of CX3CL1, which is released from the membrane, exhibits chemokine properties and acts as a chemoattractant, whereas the membrane-bound form of CX3CL1 functions as an adhesion molecule. While initial studies of CX3CL1 have demonstrated its primary role in the brain for neuron-microglia communication, subsequent research has reported that CX3CL1 expression outside the central nervous system is associated with inflammation and metabolic conditions, suggesting that CX3CL1 can be a biomarker for several diseases, such as atherosclerosis and metabolic syndrome [13,14,15,16,17].

The CX3CL1 chemokine and its sole receptor, CX3CR1 (also known as the fractalkine receptor or G-protein-coupled receptor 13 [GPCR13]), are expressed in placental and decidual tissues throughout pregnancy, and they play important roles in maternal–fetal interactions; however, the mechanisms underlying these interactions are not fully understood [18,19]. In cytotrophoblast and decidual cells, CX3CL1 is the most abundant chemokine, whereas CX3CR1 is predominantly located in placental endothelial cells and on various immune cells within the uteroplacental–fetal unit, such as monocytes/macrophages (Mφ), lymphocytes, and natural killer (NK) cells [20,21,22,23].

In the human placenta, the CX3CL12/CX3CR1 axis regulates cell adhesion, migration and angiogenesis [4,18,19]. The potential to therapeutically target these processes has been the subject of numerous studies. Dysregulation of CX3CL1/CX3CR1 signaling has been confirmed in a wide spectrum of pregnancy pathologies, from abnormal implantation and miscarriage to pregnancy-induced hypertension, gestational diabetes, fetal hypotrophy, and preterm labor [24,25,26,27,28,29].

This review presents the current state of knowledge regarding the role of CX3CL1 and its interaction with CX3CR1 in establishing placental homeostasis during placentation, and it discusses the contribution of disturbances in this interaction to placental dysfunction. These disturbances are part of the pathomechanisms of specific pregnancy complications, including PE and diabetes. This review provides a rationale for new therapeutic interventions affecting the CX3CL1/CX3CR1 axis.

## 2. Structural Duality of CX3CL1

The CX3C subclass of chemokines, which contains only CX3CL1, is characterized by the presence of three amino acids between the first two conserved L-cysteine residues (C–X3–C) within its chemokine domain [11,12,30].

The precursor compound to produce CX3CL1 is a polypeptide containing a 397-amino acid residue, including a 24-amino-acid signal peptide (SP) [31]. After synthesis is complete, the CX3CL1 protein is a single transmembrane protein whose N-terminus is on the extracellular side of the membrane (type I transmembrane protein). Mature (SP-free) transmembrane (anchored) CX3CL1 is characterized by a molecular weight of approximately 17.5 kDa, which is further increased to 95 kDa after glycosylation [11,12,32]. CX3CL1 consists of 373 amino acids, forming an extracellular N-terminal (chemokine) domain (aa 76), a mucin-like stalk (aa 241), a transmembrane α-helix (aa 19), and a short intracellular domain (aa 37) as a cytoplasmic tail [33,34,35]. The full-length membrane-bound form of CX3CL1 gives rise to a shorter soluble form of CX3CL1 (sCX3CL1) via proteolytic cleavage of the membrane-bound form near the outer surface of the membrane. Under physiological conditions, a disintegrin and metalloproteinase domain-containing protein 10 (ADAM10) is responsible for this process [33,36]. Under pathological conditions, substances rapidly released in response to stressors, such as tumor necrosis factor alpha-converting enzyme (TACE or ADAM17), matrix metalloprotease-2 (MMP-2), p38 mitogen-activated protein kinases (p38 MAPKs) α, β, γ, and δ, and lysosomal cathepsin S (CTS), may also contribute to the formation of sCX3CL1 [32,37,38]. Typically, the sCX3CL1 molecule consists of 317 amino acids and weighs approximately 14.7 kDa or 80 kDa after glycosylation, and its structure includes a chemokine domain and an extracellular mucin-like stalk [33,39,40]. The sCX3CL1 molecular weight and the number of amino acids slightly differ, which may be related to the formation of different forms of sCX3CL1 by shedding from the cell surface at alternative sites [41,42].

**Figure 1:**
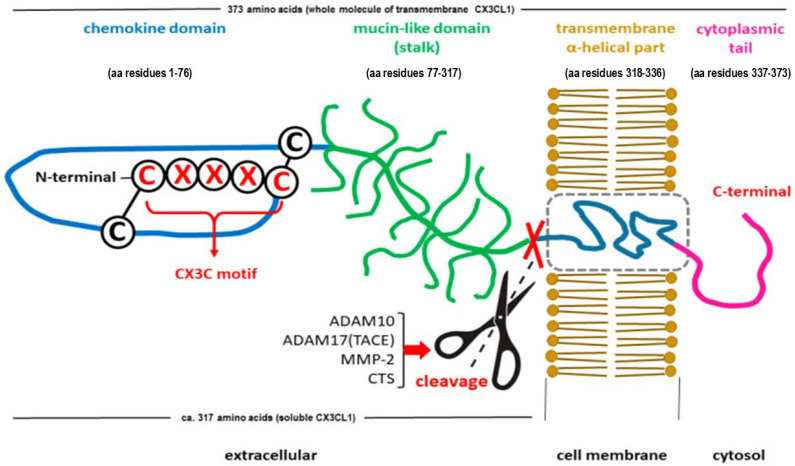
Schematic structure of the C-X3-C motif chemokine ligand 1 (CX3CL1), also known as fractalkine or neurotactin. Adapted from [14]. Both forms of the chemokine are shown: membrane-bound (transmembrane) CX3CL1 and soluble CX3CL1. The soluble CX3CL1, containing an N-terminal chemokine domain and an extracellular mucin-like stalk, is generated through the cleavage of the membrane-bound molecule near the outer surface of the membrane (marked symbolically with scissors and crossed red lines). Release of soluble CX3CL1 may occur upon exposure to a disintegrin and metalloproteinase domain-containing protein 10 (ADAM10), tumor necrosis factor alpha (TNF-α) converting enzyme (TACE or ADAM17), matrix metalloproteinase-2 (MMP-2), or cathepsins (CTS).

The unique structural duality of the chemokine CX3CL1 is shown in Figure 1.

## 3. Functional Duality of CX3CL1

Although both membrane-anchored and soluble CX3CL1 interact with the same receptor, namely, CX3CR1, these two forms of CX3CL1 translate into its functional duality [32,40], which allows CX3CL1 to act as both an adhesion molecule and a chemoattractant.

### 3.1. Membrane-Bound CX3CL1 as an Adhesion Molecule

The ability to directly bind to the cell membrane via the transmembrane domain and mucin-like stalk has been confirmed only for CXCL16 and CX3CL1 [43,44,45,46]. Owing to the increased synthesis in vascular endothelial cells, CX3CL1 gains direct access to the morphotic components of flowing blood, mainly leukocytes and platelets expressing CX3CR1 [22,47,48]. This CX3CL1–CX3CR1 interaction is particularly strong in white blood cells, and the adhesion state is characterized by a low dissociation rate, which may promote the transendothelial migration (diapedesis) of immune cells during inflammation [23,49,50].

Adhesion mediated by CX3CL1 occurs directly or as a result of combined interactions with other binding proteins found in the environment, such as cadherins, immunoglobulin superfamily cell adhesion molecules (e.g., cluster of differentiation 106 [CD106] or vascular cell adhesion protein 1, also known as vascular cell adhesion molecule 1 [VCAM-1]), selectins, and syndecans [50,51,52]. CX3CL1 may also indirectly mediate adhesion through its influence on other cellular adhesion molecules [17,53,54]. Moreover, the full adhesive strength of CX3CL1 requires these combined interactions, including CX3CL1 homo-oligomerization (forming clusters), the mucin stalk’s chemokine domain interaction with cytoskeleton to anchor and achieve adhesion stability and synergistic action with integrins (like αLβ2, αMβ2) [17,50,52].

To maintain the adhesive properties of CX3CL1, the following three conditions must be met simultaneously: (1) presentation of the CX3CL1 chemokine domain on top of a glycosylated stalk, regardless of the type of glycosylation [55,56]; (2) preservation of the unique properties of the CX3CL1 chemokine domain, which is most likely dependent on its slow receptor off-rate [57]; and (3) the optimal aggregation capacity of the transmembrane domain [54]. Similarly to that of other adhesion molecules, clustering is a sine qua non for the adhesive properties of CX3CL1. Although glycosylation of the stalk does not affect the adhesive potency of CX3CL1, it facilitates the elevation of the CX3CL1 chemokine domain fragment above the glycocalyx layer, thus enabling the ligand to target the receptor [55].

In most blood vessels, the adhesive action of CX3CL1 opposes the shear stress forces acting on the endothelium because of blood flow. Because CX3CL1 acts alone and is unable to provide optimal adhesion strength, it must act in conjunction with other adhesion molecules during leukocyte recruitment [33,49,50]. For example, the significant adhesion of leukocytes to CX3CL1 peaks at 2 dynes/cm^2^ but is minimal at a shear stress of 10 dynes/cm^2^. Unlike CX3CL1, VCAM-1 recruits leukocytes from flowing blood at 10 dynes/cm^2^. When they act together, CX3CL1 and VCAM-1 synergistically increase the number of adherent cells to double that of VCAM-1 alone [49,50].

As a multidomain transmembrane chemokine, CX3CL1 lends its chemokine domain to the CX3CR1 receptor, causing leukocyte adhesion without rolling and migration, and it also utilizes other domains crucial for its functionality by providing structural adaptation of the molecule to capture CX3CR1 in circulating cells [51,54].

The mucin stalk (mucin-like domain) stiffened by glycosylation allows the chemokine domain of CX3CL1 to be raised and maintained away from the cell membrane surface above the glycocalyx layer. Therefore, any shortening of the mucin stalk length and changes in its stiffness due to mutations in its potentially glycosylated residues may significantly impede or even eliminate the contact of the chemokine domain of CX3CL1 with leukocyte CX3CR1 [52,55]. The cytosolic domain, in turn, is responsible for adhesion strength via the cytoskeleton. The transmembrane domain, specifically the region of the transmembrane α-helix, is responsible for the monodisperse packing of CX3CL1 on the surface of CX3CR1+ cells. This region aggregates the required number of monomers to ensure adhesion and prevent rolling [55,56].

The transmembrane region of the CX3CL1 molecule mediates the dynamic balance between the plasma membrane and intracellular vesicular trafficking from the intracellular compartment. In this way, the constitutive internalization of presynthesized CX3CL1 occurs, which accelerates its cytoplasmic (intracellular) content through more rapid distribution and prevents the premature degradation of molecules by cell surface metalloproteinases [56,57]. Internalization of presynthesized CX3CL1 is based on endocytosis, and clathrin, bound by two adapter protein 2 (AP2)-binding motifs, plays a key role in the formation of the associated coated vesicles. These two motifs are YQSL, which is located at positions 362–365 within the cytoplasmic tail of amino acid residues, and YVLV, which is located between residues 392–395 (the precursor form of CX3CL1) [56]. Additionally, the properties of vesicle-associated v-soluble N-ethylmaleimide-sensitive factor attachment protein receptor (SNARE) proteins, such as syntaxin 13 (STX13) and vesicle-associated membrane protein 3 (VAMP3), determine the spatial distribution of CX3CL1 in individual subcellular compartments [58]. STX13 is known to mediate the recycling of plasma membrane proteins from endosomes, influencing the trafficking of CX3CL1 between the cell surface and endosomes, whereas VAMP3 is found on secretory granules and vesicles and participates in the continuous transport of proteins like CX3CL1 for release or insertion into the plasma membrane [58,59].

### 3.2. Chemoattractant Properties of sCX3CL1

The sCX3CL1 molecule, which is proteolytically released by metalloproteinases, contains a chemokine domain and is involved in chemotaxis, exhibiting activity similar to typical conventional chemokines. The binding of sCX3CL1 to a GPCR-type receptor on the cell surface, including CX3CR1, generates a signal that regulates the motility of chemotactic cells [31,60,61]. One of the most important roles of chemokines in the immune system is to manage the migration of leukocytes (homing) to specific anatomical locations during both inflammatory responses and normal immune homeostasis. Cells that are attracted by chemokines follow a signal of increasing chemokine concentration (concentration gradient) toward the source of the chemokine [62,63,64].

The versatility of constitutive and inflammatory response-induced CX3CR1 expression on various cells, including hematopoietic and nonhematopoietic lines, translates into various effects of CX3CL1 in the body. Among circulating blood cells, stable and increased expression of CX3CR1 is demonstrated by CD4+ T cells, CD8+ T cells, B cells (CD19+), natural killer (NK; CD56+CD3−) cells, monocytes (CD14+), thrombocytes, dendritic cells (CD11c+), mast cell (MC) progenitors and peripheral blood-derived hematopoietic stem cells (PBHSCs) [65,66,67,68,69]. In contrast, the expression of CX3CR1 is weaker in neutrophils. Owing to the weak expression of CX3CR1 in neutrophils, sCX3CL1 does not participate as a major chemoattractant in the multistep process of neutrophil transmigration across the microvascular endothelium (diapedesis) [70,71]. Conversely, membrane-anchored CX3CL1 (long form), which occurs predominantly in vascular endothelial cells, efficiently mediates the binding and adhesion of neutrophil populations, creating conditions favorable for diapedesis [72,73]. Despite the lack of strong chemoattraction for leukocytes, sensitive transendothelial migration assays have demonstrated the movement of sCX3CL1-stimulated leukocytes through the endothelium, endothelial cell chemotaxis, and the involvement of sCX3CL1 in angiogenesis, including tumorigenesis and metastasis [74,75,76,77].

### 3.3. Key Roles of CX3CL1 in the Placenta

Fractalkine plays important roles in the placenta, mediating crucial processes such as implantation, trophoblast invasion, spiral artery remodeling, angiogenesis, iron metabolism, immune cell adhesion and fetal–maternal communication at various stages of pregnancy.

**Implantation.** The CX3CL1 chemokine, which is secreted by endometrial glands, enhances endometrial receptivity by increasing the expression of receptive genes and promoting the secretion of several cytokines, such as IL-6 and IL-1β, during the process of decidualization [18,19,78,79]. These pro-inflammatory cytokines help manage the maternal immune response at the embryo-maternal interface, balancing inflammation with immune suppression needed for successful pregnancy [19,78].

**Trophoblast invasion and spiral artery remodeling.** CX3CL1 is involved in the processes of stimulating trophoblast migration, invasion and remodeling of the uterine spiral arteries into low-resistance, high-capacity vessels, which are critical for optimal placental development and, consequently, the physiological course of pregnancy [18,25,80,81].

**Angiogenesis.** CX3CL1 is also a local angiogenic factor that facilitates the formation of new blood vessels within the placenta by inducing the vascular endothelial growth factor A (VEGF-A)/VEGF receptor 2 (VEGFR-2)-related signaling pathway [17,25,26,82].

**Iron metabolism.** CX3CL1 is involved in iron metabolism at the endometrial level, the regulation of which is important for endometrial receptivity and for providing iron transport toward trophoblast cells and to the embryo [78,83,84].

**Immune cell adhesion.** The CX3CL1 chemokine mediates the adhesion of maternal immune cells to syncytiotrophoblasts, which is important for a balanced immune response at the maternal–fetal interface [16,18,19,79,85]. The CX3CR1 fractalkine receptor is expressed on several types of immune cells, including monocytes, Mφs, NK cells, dendritic cells, and various subsets of T cells (such as CD8+ effector/memory T cells, Th1 cells, and γδ T cells) [22,85,86].

**Fetal–maternal communication.** The constant, yet variable, expression of CX3CL1 in syncytiotrophoblast cells after placental development suggests the possibility of interaction between fractalkine and maternal blood cells expressing CX3CR1, which creates a specific route for communication and immune responses [18,19,23,83]. CX3CL1 therefore maintains a baseline presence for physiological interaction while fluctuating significantly in response to maternal stimuli and pathological conditions [19,81,83].

## 4. Signaling via CX3CR1 in Placental Tissue

Because the only chemokine with a CX3C motif is CX3CL1 and both membrane-bound CX3CL1 and sCX3CL1 use the same receptor, namely, CX3CR1 (previously designated V28 and also known as the fractalkine receptor of G-protein-coupled receptor 13 [GPR13]), there are favorable conditions for the interpretation of research results that focus on the C3CL1/CX3CR1 axis [31,72]. Thus, the knowledge regarding the structure of CX3CR1 and conformational changes in the receptor molecule in connection with its activation is extensive compared with the knowledge of these issues for most other cytokine receptors [87,88,89].

### 4.1. CX3CR1 Structure

In the human genome, the CX3CR1-encoding gene is located on the short arm of chromosome 3 (3p22.2), and its precise location is within the 3:39,263,495–39,281,735 genomic region on the reverse strand. *CX3CR1* has 10 transcripts (splice variants), 119 orthologs, and 23 paralogs, and it is associated with 3 phenotypes. The regulation of the genomic sequence of *CX3CR1* involves six exons (only two contain coding regions), three introns and three promoters [46,90]. Despite the different localizations of *CX3CR1* in the mouse and rat genomes [on chromosome 9 (9qF4) in mice and on chromosome 8 (8q32) in rats], human *CX3CR1* is evolutionarily conserved and contains identical or similar sequences [90,91,92].

**Figure 2:**
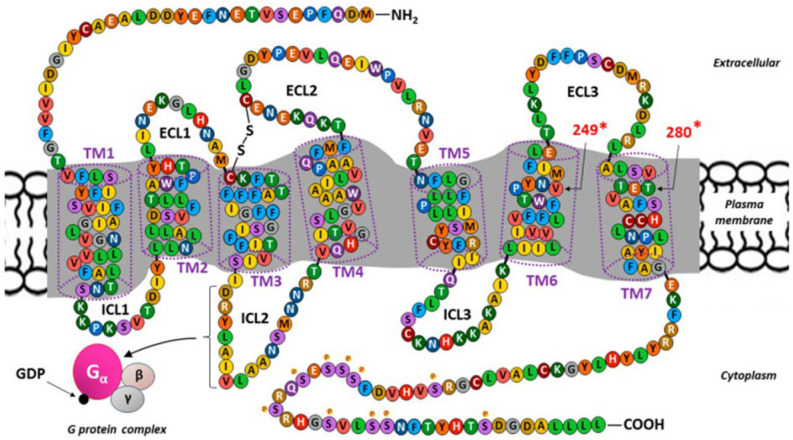
Schematic diagram and amino acid sequence of CX3C motif chemokine receptor 1 (CX3CR1), the only fractalkine (FKN, chemokine CX3CL1) receptor. Adapted from [17]. Belonging to the most numerous class A (rhodopsin-like receptors) in the G-protein-coupled receptor (GPCR) family of proteins, CX3CR1 shares a common structural signature with the polypeptide chain (355 aa; ∼40 kDa), containing seven hydrophobic α-helical transmembrane (TM1–TM7) segments or domains, with an extracellular amino terminus (NH2) and an intracellular carboxyl terminus (COOH). These transmembrane segments are connected to each other by three intracellular (ICL1–ICL3) and three extracellular loops (ECL1–ECL3) [94,95]. A disulfide bridge is marked, connecting two conservative cysteine (C) residues located at the top of the extracellular side of TM3 and within ECL2, respectively [96]. The ICL2 contains the canonical DRYLAIV motif, composed of a sequence of seven amino acids (in 3-letter abbreviations: Asp-Arg-Tyr-Leu-Ala-Ile-Val) forming a docking site that is essential for CX3CR1 coupling to the G protein and the induction of classical signaling pathways [91,97]. The binding of an agonist to CX3CR1 causes conformational changes in the receptor with the subsequent dissociation of the components of the heterotrimeric G complex, consisting of alpha (α), beta (β), and gamma (γ) subunits. Binding of guanosine diphosphate (GDP) enables the α subunit to bind to the β and γ subunits to form an inactive trimer. The binding of an extracellular signal (ligand) to CX3CR1 allows the G protein to bind to the receptor and causes GDP to be substituted by guanosine triphosphate (GTP; not shown) [93,98]. The COOH-terminal serine residues (S) are susceptible to G-protein-coupled receptor kinase (GRK)-mediated phosphorylation (marked with tiny yellowish dots containing P). * The polymorphic residues at positions 249 and 280 may be responsible for dysfunctional CX3CR1 variants [99,100].

Both the 355-amino-acid sequence and the three-dimensional structure of the transmembrane molecule (MW∼40 kDa) corresponding to CX3CR1 are well known [72]. The membership of CX3CR1 among the metabotropic receptors, or more precisely, the most numerous class A (rhodopsin-like receptors) in the GPCR protein family, is characterized by a distinctive structure. CX3CR1 consists of a monomeric protein containing an extracellular domain with a signaling ligand binding site and an intracellular domain binding the G protein [93]. The amino acid sequence of CX3CR1 and its structure as a seven-transmembrane receptor (7-TM) are shown in Figure 2.

The CX3CR1 polypeptide chain in the form of seven α-helical structures crossing the cell membrane forms a spatial arrangement composed of transmembrane segments (TM1–TM7) and eight amino acid chains on both sides of the membrane, which connect individual TMs, including three extracellular loops (ECL1–ECL3), three intracellular loops (ICL1–ICL3), and two linear chains terminated by an extracellular amino terminus (NH2) and an intracellular carboxyl terminus (COOH) at the respective ends of the molecule [35,43]. Two conserved cysteine (C) residues in the CX3CR1 molecule are bound by a disulfide bond that connects the extracellularly located TM3 vertex and ECL2 [96]. Binding sites for functional ligands, such as CX3CL1 and CCL26 (eotaxin-3), as well as antibodies and some bacterial and viral pathogens, are located within the ECLs and NH2 terminus [101,102,103,104]. Most GPCR chemokine receptors, including CX3CR1, require an appropriate level of tyrosine (T) sulfation at the NH2-terminus to maintain optimal activity [32,105].

In the cytoplasm, on the other side of the cell membrane, ICL2 plays a crucial functional role because of the presence of seven amino acids that make up the canonical DRYLAIV motif. This sequence, given as three-letter abbreviations, corresponds to Asp-Arg-Tyr-Leu-Ala-Ile-Val [91,97]. The presence of this amino acid arrangement provides a docking point that is required for proper coupling of CX3CR1 to a G protein belonging to the Gαi family. Because the structure of metabotropic receptors does not contain ion channels, such CX3CR1/Gαi coupling is necessary for the induction of classical signaling pathways via ion flow associated with the activation of an intermediary G protein [32,106].

Upon agonist binding, conformational changes in the CX3CR1 molecule occur, and consequently, dissociation of the alpha (α), beta (β), and gamma (γ) subunits, which are components of the heterotrimeric G complex, occurs. Binding of guanosine diphosphate (GDP) allows the α subunit to bind the β and γ subunits, resulting in the formation of an inactive trimer (inactive Gα-GDP state). The conversion of the inactive trimer into the active form requires the replacement of GDP with guanosine triphosphate (GTP), which occurs upon the release of an extracellular signaling molecule (ligand). Active Gα-GTP is then formed, enabling the G protein to bind to CX3CR1 [43,106,107].

G protein-coupled receptor kinases (GRKs) cause the desensitization of CX3CR1 by phosphorylating serine (S) residues within the COOH-terminus. However, as subsequent studies have shown, GRKs perform additional functions as scaffolds and signaling adapters, which broadens the range of physiological competences of this small family of proteins in modulating CX3CR1 activity [107,108].

Like other chemokine receptors, CX3CR1 is polymorphic and may be responsible for its varying affinity for CX3CL1 and other ligands [109]. Dysfunctional variants of CX3CR1, including those observed in various cancers, may be accompanied by polymorphic residues at positions 249 and 280 [99,100]. Polymorphism of CX3CR1 has also been linked to infections (e.g., systemic candidiasis), diseases of the circulatory system (e.g., atherosclerosis) and the nervous system (e.g., Alzheimer’s disease) [110,111,112,113].

### 4.2. Main Signaling Pathways Involved in the CX3CL1/CX3CR1 Axis in the Placenta

**Figure 3:**
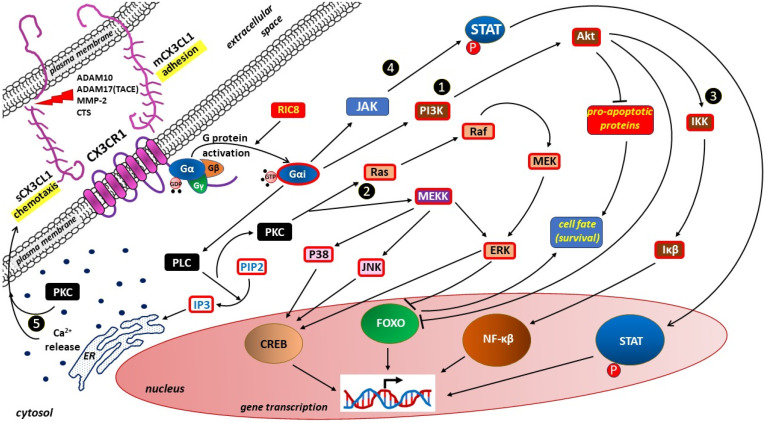
The most important signaling pathways within the chemokine CX3CL1 (fractalkine)/CX3C motif chemokine receptor 1 (CX3CR1) axis. Diagram simplified for clarity; interactions with other receptors and signaling pathways, including mammalian target of rapamycin (mTOR), toll-like receptors (TLRs), Wnt/β-catenin, and Ras homolog gene family member A (RhoA) signaling pathways, are omitted. Both forms of the only endogenous CX3CR1 ligand, i.e., membrane bound CX3CL1 (mCX3CL1) and—resulting from membrane shedding by lysosomal protease cathepsin S (CTS) and/or metalloproteases (a disintegrin and metalloproteinase domain-containing protein 10 [ADAM10], a disintegrin and metalloprotease 17 [ADAM17], also called tumor necrosis factor alpha converting enzyme, [TACE], and matrix metalloprotease-2 [MMP-2])—soluble CX3CL1 (sCX3CL1), activate the same signaling pathways promoting adhesion or chemotaxis, respectively. Chemotaxis is a primary mechanism driving trophoblast invasion, while cell adhesion profoundly influences cell proliferation and differentiation, being also a fundamental regulator of angiogenesis. During G protein activation, resistance to inhibitors of cholinesterase 8 homolog A (RIC8) acts as a non-receptor guanine nucleotide exchange factor (GEF), catalyzing the release of guanosine diphosphate (GDP) from inactive Gα subunit and allowing guanosine triphosphate (GTP) to bind and activate the G protein. Binding of GTP to the Gα molecule of the G protein complex causes dissociation to form Gαi-GTP and a GβGγ dimers. Activated Gαi interacts with various downstream effectors causing signaling within ❶—phosphoinositide 3-kinase (PI3K)/protein pinase B (Akt) pathway; ❷—mitogen-activated protein kinase/extracellular signal-regulated kinase 1/2 (MAPK–ERK1/2) pathway; ❸—nuclear factor kappa-light-chain-enhancer of activated B cells (NF-κB) pathway; ❹—Janus kinases (JAK)/signal transducer and activator of transcription (STAT) proteins pathway; and ❺—intracellular calcium (Ca^2+^) release from the endoplasmic reticulum (ER). Taking place via the phospholipase C (PLC)/protein kinase C (PKC) pathway, intracellular divalent calcium cations (Ca^2+^) mobilization is a critical second messenger in chemotaxis. Ultimately, these signaling pathways lead to changes in gene transcription through activation of the STAT, NF-κβ and cAMP/Ca^2+^ response element binding protein (CREB), while inhibiting the members of the class O of forkhead box transcription factors (FOXO). By inhibiting the expression of pro-apoptotic proteins and FOXO activity, signaling pathways via PI3K/Akt/IkappaBeta (Iκβ) kinase (IKK)/Iκβ/NF-κβ pathway, Ras kinases (Ras)/Raf kinases (Raf)/mitogen-activated protein kinase kinase(MEK)/extracellular signal-regulated kinase (ERK) and MEK kinase (MEKK)/cJun NH(2)-terminal kinase (JNK)/CREB or MEKK/mitogen-activated protein kinases (P38)/CREB pathways can increase the survival of cells. Other abbreviations: Gα, Gβ, Gγ—subunits of the heterotrimeric G-proteins (G protein complex); Gαi—activated Gα subunit of the G protein complex; IP3—inositol 1,4,5-trisphosphate; PIP2—phosphatidylinositol 4,5-biphosphate; p—phosphorylation.

Binding of each of the two forms of CX3CL1 to CX3CR1 leads to the activation of heterotrimeric G proteins with subsequent recruitment and activation of several downstream signaling cascades, which influences the cellular response by modulating gene expression [17]. Angiogenesis, resistance to apoptosis, cell migration and proliferation, among other processes, are controlled by this mechanism [17,35]. The most important signaling pathways within the CX3CL1/CX3CR1 axis are presented in Figure 3.

#### 4.2.1. Phosphoinositide 3-Kinase/Protein Kinase B Pathway

The phosphoinositide 3-kinase (PI3K)/protein kinase B (Akt) pathway is highly conserved, and its activation is tightly controlled by a multistep process [114]. This is because activation of the PI3K/Akt pathway influences fundamental cellular processes, including protein synthesis, cell survival, proliferation, and resistance to apoptosis [115,116,117]. PI3K activation is mediated by the binding of agonistic ligands to a wide range of receptors belonging to different classes, such as receptor tyrosine kinases (RTKs), integrin receptors, and cytokine receptors, including some 7-TM receptors, such as CX3CR1 [118,119,120,121].

Under the influence of PI3K, phosphatidylinositol (3,4,5)-trisphosphate (PIP3) is formed, which is responsible for the translocation of Akt to the cell membrane and enables the activation of Akt through its phosphorylation at the Thr308 residue involving phosphoinositide-dependent kinase 1 (PDK1) [122,123,124]. In addition to its crucial role in the regulation of insulin signaling and glucose metabolism, Akt mediates various downstream signaling pathways of PI3K, such as promoting protein synthesis via the activation of mammalian target of rapamycin (mTOR) and p70S6 kinase (p70S6K, also known as ribosomal protein S6 kinase beta-1 [S6K1]), attenuating proapoptotic signals by inhibiting the B-cell lymphoma 2 (BCL2)-associated agonist of cell death (BAD) protein or controlling autophagy by interacting with gamma-aminobutyric acid (GABA) receptors [116,117,125,126,127,128]. In this way, by activating the PI3K/Akt pathway and acting on GABA receptors, the CX3CL1/CX3CR1 axis may indirectly influence the course of pregnancy, especially during its initial period. In humans, GABAergic transmission, through the regulation of human chorionic gonadotropin (hCG) secretion, is involved in the control of the hormonal activity of the corpus luteum. Activation of the GABA-A receptor stimulates hCG secretion. Additionally, signaling via the GABA-A receptor π subunit (GABRP) reduces trophoblast invasiveness and increases apoptosis [126,129,130].

Optimal signaling levels through the PI3K/AKT, Janus kinase-signal transducer and activator of transcription (JAK/STAT), and estrogen signaling pathways, along with interactions with cell surface proteins that bind to extracellular matrix (ECM) receptors, determine proper placental cotyledon development with appropriate development of the villous network and capillary density [131]. The spectrum of signaling through the above pathways is crucial for cell survival by regulating apoptosis and includes activation of cytokines and growth factors vital for growth, angiogenesis, proliferation, and migration, including trophoblast invasion and maternal vessel transformation during placentation [131].

The expression of matrix metalloproteinases (MMPs), members of the zinc-dependent ECM remodeling endopeptidases family, such as MMP1, MMP12, MMP14, and MMP15 is also regulated via the PI3K/AKT pathway. ECM degradation and tissue reorganization are crucial for placental vascularization and embryonic development [132,133,134]. Membrane-type matrix metalloproteinases (MT-MMPs), which are members of the MMP subfamily, play an important role during pregnancy, and PI3K/AKT signaling ensures precise control of their activity in time and space [134]. Such activity is required for cytotrophoblast migration and invasion of the uterine wall and in the remodeling of the spiral arteries. MT-MMPs are involved in the fusion of cytotrophoblasts to form syncytiotrophoblasts and in angiogenesis [134,135].

#### 4.2.2. Mitogen-Activated Protein Kinase–Extracellular Signal-Regulated Kinase 1/2 Pathway

The mitogen-activated protein kinase (MAPK)–extracellular signal-regulated kinase 1/2 (ERK1/2) pathway is among the main signaling pathways that translates extracellular signals, such as those related to growth factors, into a wide range of cellular responses, including proliferation, differentiation, stress responses and survival (through the regulation of programmed cell death) [136,137].

Placental tissue studies have shown that appropriate MAPK–ERK signaling in specific cell types is essential for ensuring proper morphogenesis during the growth and maturation of the placenta [138]. During placental formation, the MAP2K1 and MAP2K2 genes play important roles in the development of the placental labyrinth, vascularization and blood-placental barrier formation, and they are responsible for encoding dual-specificity kinases responsible for ERK activation, namely, mitogen-activated protein kinase kinase 1 (MEK1) and mitogen-activated protein kinase kinase 2 (MEK2), respectively [138,139,140]. In mice, loss of *MAP2K1* function causes embryonic lethality because of placental defects, whereas *MAP2K2* mutants survive with a normal lifespan, suggesting that *MAP2K1* masks the phenotype because of the *MAP2K2* mutation [140]. Nevertheless, isolated deletion of *MAP2K2* results in altered expression of several genes involved in apoptosis, cell fusion and cell polarity in syncytiotrophoblasts during placentation [140].

The MAPK–ERK1/2 axis is also a target of the endogenous high-affinity 54-amino acid apelin receptor agonist called Elabela (ELA) [141,142]. After being enzymatically cleaved, the ELA molecule contains 32 amino acids (ELA-32) and is strongly expressed in both cytotrophoblasts and syncytiotrophoblasts of the placenta [141]. ELA significantly increases trophoblast invasion into uterine spiral arteries, promoting vascular remodeling to create a low-resistance vascular bed in early pregnancy [142].

Immunolocalization studies of ERK1/2 in the human placenta have revealed that MAPK–ERK1/2 pathway activity is limited to the first trimester of gestation [143]. Phosphorylated (active) ERK1/2 is immunolocalized in villous cytotrophoblasts (VCTs) only up to 12 weeks of gestation [143]. Similarly, c-MET, a receptor tyrosine kinase that binds hepatocyte growth factor (HGF; also known as scatter factor) and utilizes the MAPK–ERK1/2 signaling pathway, is highly expressed in VCTs until the end of the first trimester [144]. HGF acts via the c-MET receptor, resulting in pronounced effects on certain epithelial cells [145]. Given that the trophoblast is a specialized, epithelial-origin barrier that forms a critical part of the placenta, the way in which the expression of both c-MET and HGF changes during pregnancy suggests their involvement in trophoblast transformation in early pregnancy [144,145].

ERK1/2 and p38 mitogen-activated protein kinases (p38MAPKs), a class of MAPKs, play significant roles in initiating trophoblast differentiation and fusion [146]. Epidermal growth factor (EGF) stimulates cytotrophoblast fusion via the p38MAPK pathway [146,147]. Although p38MAPK is a kinase that is responsive to stress stimulation, invasive trophoblast cells do not express the activated form of p38 MAPK [145,146].

#### 4.2.3. Nuclear Factor Kappa-Light-Chain-Enhancer of Activated B Cells Pathway

Signaling related to the activation of the nuclear factor kappa-light-chain-enhancer of activated B cells (NF-κB) pathway by the CX3CL1/CX3CR1 axis leads to the nuclear translocation of transcription factors and the expression of proinflammatory or anti-inflammatory genes, depending on the context [148,149].

The NF-κB pathway in the human placenta is a critical signaling pathway involved in placental development, implantation, spiral artery remodeling, and the regulation of the onset of labor [150,151]. NFκB is activated by the inflammation and hypoxia that occurs in early pregnancy [152]. Oxidative stress and angiogenesis are inextricably linked to trophoblast invasion and placental development, in which signaling via the NF-κB pathway plays a leading role [152,153]. By influencing the secretion of cytokines, growth factors, and/or hormones, NF-κB also plays a pivotal role in the physiology and pathophysiology of labor [154].

Increased placental signaling through the NF-κB pathway is often associated with placental dysfunction (see Section 5; Placental dysfunction and CX3CL1/CX3CR1 signaling).

#### 4.2.4. JAK/Signal Transducer and Activator of Transcription Proteins Pathway

STAT proteins are pivotal regulators of signaling cascades that communicate information from chemical signals outside of a cell to the cell nucleus. The JAK/STAT signaling pathway is involved in modulating gene expression and immune responses, and it influences cell division, cell death, cancerous transformation and tumor growth [155]. CX3CL1/CX3CR1 and JAK/STAT signaling pathways complement each other in mediating inflammation-related processes, including those specific to the placenta and those related to implantation, trophoblast invasion, and angiogenesis [18,93,156,157]. Additionally, the development of maternal–fetal immunotolerance requires mutual interactions within both signaling axes, the functionality of which should be considered together [158,159].

JAK/STAT activity is essential for proper endometrial decidualization [160,161]. At the early stage of pregnancy, mainly under the influence of progesterone and estradiol, the ERK1/STAT3 signaling pathway is activated in endometrial stroma cells, which subsequently promotes the expression of the CCAAT/enhancer binding protein β (C/EBPβ) transcription factor [162,163]. The above processes and the secretion of prolactin (PRL) by decidual cells stimulate the decidualization of human endometrial stromal cells with interleukin IL-6- and IL-11-dependent activation of STAT3 [164,165]. Additionally, activation of STAT3 signaling in decidual cells is induced by leukemia inhibitory factor (LIF), which enhances endometrial receptivity by promoting the expression of early growth response 1 (EGR1) protein [166,167]. Endometrial receptivity for pregnancy also increases in the presence of growth hormone (GH) in placental tissue, which activates the JAK2/STAT5 signaling pathway in decidual cells, leading to an increase in the expression of integrin β3 [168]. Constantly increased STAT5 expression may facilitate the decidualization process by enhancing PRL expression in human endometrial stromal cells [169]. In summary, the CX3CL1/CX3CR1 axis is involved in the preparation of the endometrium for blastocyst implantation, and in the process of the transformation of endometrial stromal cells into specialized decidual cells, it cooperates extensively with JAK/STAT signaling [79].

The interferon-γ (IFN-γ)-activated JAK/STAT1 signaling pathway may participate in the CX3CL1-induced delocalization of intercellular E-cadhedrin from the cell–cell junctions to the cytoplasm, which accompanies spiral artery remodeling and may prevent excessive migration and invasion of trophoblasts [27,170,171].

Under physiological conditions, CX3CL1/CX3CR1 signaling leads to the development of increased immune tolerance, which prevents the onset of inflammatory immune responses that may cause fetal rejection [18,19]. Following the recruitment of immune cells to the maternal–fetal interface at the decidua, decidual dendritic cells (DCs), natural killer cells (NKs), Mφs and T cells interact with trophoblast cells, and trophoblast function is modulated after the activation of JAK/STAT signaling [80,159].

STAT1 and STAT3 inhibit decidual Treg cell differentiation, which may have unfavorable consequences for pregnancy by enhancing the immune response, whereas STAT5 promotes immune tolerance by enhancing decidual Treg differentiation and inhibiting Th1 differentiation, which consequently limits the activation of Mφ and cytotoxic T cells at the site of implantation [172,173,174]. STAT3 signaling may also promote pregnancy development, as STAT3 functions in myeloid-derived suppressor cells (MDSCs), which are crucial for establishing maternal–fetal immune tolerance and promoting successful implantation in the first trimester of a healthy pregnancy [175,176]. In human MDSCs, major histocompatibility complex, class I, G (HLA-G), a molecule that functions primarily as an immune checkpoint to promote immune tolerance during pregnancy by inhibiting the activity of immune cells (such as NK cells, T cells, and B cells), binds to the Ig-like transcript 4 inhibitory (ILT4) receptor to activate STAT3 [177,178]. STAT3 then promotes the expression and secretion (activity) of indoleamine 2,3-dioxygenase 1 (IDO1), a key enzyme for preventing maternal immune rejection of the semiallogenic fetus by breaking down tryptophan, thereby reducing the availability of this amino acid for T cells [179,180].

JAK/STAT signaling also influences Mφs to obtain functional characteristics optimal for maintaining pregnancy. Activation of STAT3 promotes anti-inflammatory M2 polarization, whereas inhibition of STAT1 and STAT5 activity counteracts proinflammatory M1 polarization [159].

#### 4.2.5. Intracellular Calcium Release

CX3CL1 binding to CX3CR1 induces intracellular Ca^2+^ release in cells expressing the receptor, as the CX3CR1 receptor is a metabotropic GPCR that activates associated signaling pathways, including those linked to calcium signaling [181]. Because trophoblast cells, particularly certain invasive types, and maternal decidual cells—especially decidual stromal cells (DSCs) and immune cells within the decidua, such as decidual Mφ, NK cells, and T cells—express CX3CR1, the involvement of calcium ions in placental signaling via CX3CL1/CX3CR1 is evident [19,78,85].

Activation of CX3CR1 involves the nucleotide exchange of guanosine-5′-triphosphate (GDP) for guanosine-5′-diphosphate (GTP), followed by dissociation of the activated alpha subunit (Gαi) from the G protein Gαβγ heterotrimer [17]. This initiates a signal transduction cascade, the first step of which is the activation of phospholipase C (PLC). PLC then cleaves phosphatidylinositol 4,5-bisphosphate (PIP2) within the plasma membrane, producing two critical second messengers, namely, inositol 1,4,5-trisphosphate (IP3) and diacylglycerol (DAG). DAG remains in the membrane to activate protein kinase C (PKC), which triggers diverse cellular responses through the phosphorylation of target proteins, while soluble IP3 diffuses into the endoplasmic reticulum (ER) and activates intracellularly stored calcium, shifting Ca^2+^ into the cytoplasm [181,182,183]. Increased cytoplasmic Ca^2+^ concentration, a central component of signal transduction, leads to chemotaxis and initiates widespread cellular responses in placental tissue, which are crucial for successful embryo implantation and regulate trophoblast migration, invasion, angiogenesis, and overall fetal–maternal interactions, influencing pathways for growth and survival, although expression levels vary by cell line and gestational stage [19,26,184]. However, specific details on calcium-handling proteins and calcium release from placental cells still need more in-depth research [185,186,187,188].

#### 4.2.6. Other Pathways

The CX3CL1/CX3CR1 axis also interacts with mammalian target of rapamycin (mTOR), toll-like receptors (TLRs), Wnt/β-catenin, and Ras homolog gene family member A (RhoA) signaling pathways, among others [17,189,190,191,192]. The importance of this signaling at the level of placental tissue and at different stages of pregnancy needs further research.

## 5. Placental Dysfunction and CX3CL1/CX3CR1 Signaling

Because the CX3CL1/CX3CR1 axis and other signaling pathways indirectly related to it play a vital role in regulating implantation and placentation (including vascular development), as well as are involved in the development of maternal–fetal immune tolerance and in the mechanisms triggering labor, the placenta is a central interface for these activities [19,80,85,193].

Placental dysfunction may therefore be a consequence of altered endometrial receptivity and embryo implantation, and it may be associated with suboptimal trophoblast proliferation, differentiation and migration [194]. This may result in insufficient invasion of extravillous trophoblasts into the maternal uterine spiral arteries and disruption of their remodeling from high-resistance vessels into wide, low-resistance channels. This form of abnormal placentation is part of the pathomechanism of PE, a pregnancy disorder characterized by hypertension and proteinuria in the mother, which are derivatives of shallow trophoblast invasion with an ischemic placenta that triggers widespread endothelial dysfunction, inflammation, vasoconstriction, and immune dysregulation [195,196].

Another frequently occurring complication of pregnancy, in which the pathomechanism of CX3CL1/CX3CR1 signaling in a dysfunctional placenta plays an important role, is gestational diabetes mellitus (GDM). GDM causes placental dysfunction by inducing inflammation, oxidative stress, and abnormal vascularization, leading to various structural changes, such as villous immaturity, poor nutrient/oxygen exchange, and altered placental development [26,197,198].

Placental dysfunction accompanying PE and GDM covers the entire range of activities controlled by the CX3CL1/CX3CR1 axis.

### 5.1. Dysregulated Placental Fractalkine Signaling in Preeclampsia

Unlike pregnancy-induced hypertension, which is characterized by new high blood pressure (≥140/90 mmHg) developing after 20 weeks of pregnancy, preeclampsia (PE) is a serious pregnancy-specific condition involving high blood pressure at or beyond 20 weeks of gestation with proteinuria and/or evidence of new end-organ (such as kidney, liver, and brain) dysfunction, potentially leading to seizures (eclampsia) or hemolysis, elevated liver enzymes and low platelet (HELLP) syndrome [199].

Even in normal pregnancy, a mild systemic inflammatory response occurs at the uteroplacental level, which tends to gradually increase with gestational age, peaking in the third trimester and perinatal period [200,201]. The proinflammatory component inherent in the establishment of immunotolerance in the maternal–fetal system is also evident at the placental level through increased circulating inflammatory cytokines and the activation of lymphocytes, granulocytes, and monocytes [202,203]. In the cytokine milieu, the CX3CL1 chemokine plays an important role [17].

Compared with that in normotensive pregnancies, the intensity of the systemic inflammatory response clearly increases in PE pregnancies, as evidenced by, among other characteristics, a change in the cytokine profile, including significantly increased levels of proinflammatory maternal circulating cytokines [204,205]. Gene expression analysis, immunohistochemistry and enzyme-linked immunosorbent assays (ELISAs) have confirmed that the expression levels of both CX3CL1 and CX3CR1 are significantly greater in the placental tissues and decidual Mφs of patients with severe PE than in those of patients with healthy pregnancies [20,23,206]. Moreover, these changes are accompanied by significantly increased expression of the ADAM10 and ADAM17 metalloproteinases, which are involved in the conversion of membrane-bound CX3CL1 to sCX3CL1 [206].

Studies in which first-trimester placental explants were incubated with TNF-α have suggested that increased maternal TNF-α may upregulate the expression and release of placental CX3CL1, which in turn may contribute to an exaggerated systemic inflammatory response in PE [206]. Thus, a vicious cycle of disease is created [207].

Because CX3CL1 mediates the migration, survival, and adhesion of monocytes, NK cells, and T cells to endothelial/trophoblast cells, any abnormalities in the abundance of these immune cells in the decidua may elicit PE development [208,209]. Studies on incubated primary human first-trimester decidual cells (FTDCs) have shown that stimulation with increased concentrations of decidual Mφ-derived TNF-α and interleukin-1 β (IL-1β), as well as NK cell-derived interferon-γ (IFN-γ), may be responsible for the significant increase in CX3CL1/CX3CR1 signaling in the disturbed proinflammatory cytokine profile [20]. IL-1β-, TNF-α-, and IFN-γ-induced CX3CL1 production in FTDCs occurs after the activation of various signaling pathways, including MAPK–ERK1/2, JAK/STAT, and NFκB [20]. Moreover, decidual/placental cell-secreted CX3CL1 is involved in the later development of PE, whereas circulating CX3CL1 levels do not predict PE [20].

Significant underdevelopment of the placental vascular network has been demonstrated in PE, especially in pregnancies complicated by FGR [210]. The CX3CL1/CX3CR1 signaling pathway is involved in the pathomechanism of this defective development of the placental vasculature [25]. Under physiological conditions, the angiogenic potential of the CX3CL1 chemokine is revealed after activation of CX3CR1 by a two-step mechanism involving hypoxia-inducible factor 1 alpha (HIF-1α) and VEGF and by stimulation of integrin-dependent trophoblast migration, the key point in the process of trophoblast invasion toward spiral arteries [80,82]. Hypoxia is also an independent stimulator of both CX3CL1 synthesis and CX3CR1 expression in placental tissue [21]. In the serum of pregnant women collected during the third trimester, the concentration of sCX3CL1 is significantly greater in preeclampsia pregnancies than in normal pregnancies, and this increase is accompanied by increased expression of CX3CR1 in syncytiotrophoblasts but low expression of CX3CR1 in the endothelium within the placenta [25]. A comparative study of vascular density in placental samples (PE vs. normal pregnancy) has reported that the lowest vascular/extravascular tissue index (V/EVTI) values are in PE, especially when complicated by FGR, indicating impaired angiogenesis in PE [25]. In this case, increased CX3CR1 receptor density and elevated CXCL1 concentrations in serum and placental tissue in PE may suggest an ineffective proangiogenic effect of the compensatory mechanism associated with CX3CL1/CX3CR1 signaling [25]. The appearance of CX3CR1 on syncytiotrophoblastic cells with concomitant low expression in the endothelium in preeclamptic placentas confirms the general pathogenesis of preeclampsia, which is a type of dysfunctional angiogenesis [211].

### 5.2. Upregulated Expression of the CX3CL1/CX3CR1 Axis in Diabetic Placenta

Glycemic control disorders observed during pregnancy may be associated with preexisting type 1 diabetes (little to no insulin), type 2 diabetes (insulin resistance), or GDM, when hyperglycemia develops or is first diagnosed during pregnancy (typically in the second or third trimester) [212,213]. Approximately 14% of pregnancies globally are affected by GDM, and the prevalence of GDM has increased globally in the last 20 years by approximately 15–100%, owing to increasing obesity, older maternal age, lifestyle changes (e.g., less physical activity), and shifting diagnostic criteria [214,215,216]. Diabetic pregnancy complications affect both the mother and the fetus, including higher risks for PE, miscarriage, preterm birth, cesarean sections, and infections for the mother, while the fetus/newborn faces risks such as macrosomia resulting in excessive birth weight, birth defects (heart, brain, or spine), breathing problems, jaundice, postnatal hypoglycemia and future obesity/type 2 diabetes [217].

During pregnancy complicated by diabetes, significant morphological and functional changes occur in the placenta, the intensity of which is usually directly proportional to the severity of metabolic disorders associated with hyperglycemia and therefore inversely proportional to the adequacy of blood glucose control [198,218,219].

Research is being undertaken to determine the extent to which CX3CL1/CX3CR1 signaling contributes to the development of placental structure and functional disorders in GDM [197,220,221]. Attention is given to the altered immune response, especially related to the innate immune response [222,223,224]. As essential components of the innate immune system, Mφs play key roles in successful placentation and fetal development, and their proper functional polarization has been shown to be crucial [225,226]. In hyperglycemia accompanying GDM, Mφ reprogramming occurs, which, in response to signals accompanying metabolic disorders, including an altered cytokine profile, promotes proinflammatory polarization (M1) to a greater extent than anti-inflammatory polarization (M2) [227]. The altered M1/M2 ratio in the GDM placenta may be due to upregulation of the CX3CL1/CX3CR1 axis through the NF-κB signaling pathway, which promotes M1-type macrophage polarization [44,227]. Such upregulation of the CX3CL1 chemokine has been reported in the capillary endothelium and human umbilical vein endothelial cells (HUVECs) when comparing placentas obtained after pregnancies complicated by GDM with placentas from normal pregnancies [197].

Diabetic pregnancies exhibit increased placental CX3CL1 expression and increased placental microvessel density, a key feature of diabetic placentopathy, suggesting that the CX3CL1/CX3CR1 pathway is involved in the pathological remodeling of the placental microvasculature under these conditions [23]. Comparative studies of placental sections have compared diabetes class C complicated pregnancies with normal pregnancies. The class C diabetes in pregnancy (after White) refers to diabetes that developed before pregnancy, between the ages of 10 and 19 years or between the durations of 10 and 19 years, and it is the last stage without recognized vascular morphological changes on light microscopy [228,229]. An evaluation of the relationship between the mean CX3CR1 expression and mean V/EVTI in placental samples obtained from the maternal surface of the placenta has also revealed a strong positive correlation and significant differences between the groups. Moreover, an immunohistochemical technique for the identification of CX3CR1 has revealed that this receptor is predominantly located in placental endothelial cells. Therefore, the increased endothelial expression of CX3CR1 in individuals with diabetes corresponds to increased placental vascularization, as assessed by the V/EVTI [26].

Because the induction of a proinflammatory placental environment is inherently involved in the pathophysiology of diabetic pregnancy, it can be assumed that the intensification of local angiogenesis occurs as a result of frequent episodes of local hypoxia and transient hyperglycemia (even in well-controlled cases), which are associated with elevated levels of reactive oxygen species (ROS), advanced glycation end products (AGEs), and some proinflammatory cytokines, especially those with angiogenic properties [230,231,232,233,234,235]. Increased levels of TNF-α, IFN-γ, and IL-1β in response to hypoxia may result in increased CX3CL1 gene expression and increased CX3CL1 levels with subsequent angiogenesis via the PI3K-PKB/Akt/eNOS-dependent pathway and MAPK–ERK1/2 signaling [236,237].

In the placentas of diabetic patients, increased expression of MMPs and ADAMs, including ADAM17/TACE or sheddases, has been reported; this increased expression may result in an increase in the concentration of sCX3CL1, which is derived from its membrane form. Considering the chemoattractant activity of sCX3CL1, increased migration of a specific CX3CR1-positive subpopulation of inflammatory cells, including mast cells (MCs), may occur in placental tissue [238,239]. An increased number of MCs in the diabetic placenta may be responsible for the change in the cytokine profile toward a proinflammatory state and may also be associated with increased MC-dependent angiogenesis [240,241,242,243]. Notably, CX3CL1 does not directly participate in MC-dependent angiogenesis because despite the expression of CX3CR1 on the surface of MCs, it does not promote MC degranulation on its own [70].

In terms of the increased endothelial expression of CX3CR1 in the diabetic placenta, CX3CL1 production induced by various factors is also subject to autoregulation through modulating the expression of the CX3CR1 receptor [29,244]. The existence of autoregulation between CX3CL1 and CX3CR1 via an autocrine loop (CX3CL1/CX3CL1 axis) has been suggested in independent studies with respect to many cell types, including endothelial cells [244,245,246].

## 6. Concluding Remarks

The CX3CL1 chemokine (fractalkine), the only known member of the CX3C chemokine family, has unique properties and performs various functions, and it exists in two forms—an adhesion molecule (membrane-bound CX3CL1) and a chemoattractant (sCX3CL1). Therefore, membrane-bound CX3CL1 captures, holds and activates immune cells (monocytes, Mφs, T cells, and NK cells), whereas sCX3CL1 attracts these cells to guide cell movement. CX3CL1 is abundantly expressed in placental tissue, where its levels change according to the stage of pregnancy and the presence of certain pregnancy complications, including PE and diabetes. Together with the only known receptor, CX3CR1, CX3CL1 forms a signaling axis that is deeply interconnected and influences numerous other pathways that control inflammation, immune tolerance, angiogenesis, apoptosis resistance, cell migration and proliferation. Because key sites of CX3CL1 expression include decidual tissue, syncytiotrophoblasts within the apical microvillous plasma membrane, and the endothelial cells of placental capillaries, proper CX3CL1/CX3CR1 signaling ensures optimal immune homeostasis and cytokine profiles. CX3CL1/CX3CR1 signaling acts as a critical regulator of maternal-fetal crosstalk and orchestrates key stages of pregnancy from initial blastocyst implantation through placentation to the final placental maturation and onset of labor.

Changes in the functioning of the placental CX3CL1/CX3CR1 axis are involved in the pathomechanism of low-grade inflammatory background and endothelial dysfunction in both GDM and PE, and the risk of developing PE during GDM is inversely proportional to the degree of the correction of metabolic disorders associated with hyperglycemia. Therefore, the possibility of therapeutically influencing the activity of the CX3CL1/CX3CR1 axis in the case of placental dysfunction accompanying PE and diabetes-complicated pregnancy is the subject of ongoing research. Direct interference with signaling pathways using newly developed active compounds may be a strategy to improve pregnancy outcomes, although one cannot overlook the potential side effects of such treatments, including teratogenic ones, on the fetus. The therapeutic implications must also consider the difficulties associated with ensuring selective effects on CX3CL1/CX3CR1 signaling within the placenta or the maternal–fetal interface that would not lead to systemic effects, for example, on the central nervous system.

## Data Availability

No new data were created or analyzed in this study. Data sharing is not applicable to this article.

## Abbreviations

7-TM receptor	seven-transmembrane receptor
ADAM10	a disintegrin and metalloproteinase domain-containing protein 10
ADAM17	a disintegrin and metalloproteinase domain-containing protein 17, also known as tumor necrosis factor alpha converting enzyme (TACE)
AGEs	advanced glycation end products
Akt	protein kinase B
AP2	adapter protein 2
BAD protein	B-cell lymphoma 2-associated agonist of cell death protein
C/EBPβ	CCAAT/enhancer binding protein β–transcription factor
CD	chemokine domain
CD106	cluster of differentiation 106, an adhesion molecule
c-MET	receptor tyrosine kinase
CTS	cathepsin S
CX3CL1	chemokine C-X3-C motif ligand 1, also known as fractalkine (FKN)
CX3CR1	ligand 1 (CX3CL1) receptor, also known as G-protein-coupled receptor 13 (GPR13), previously known as V28
DAG	diacylglycerol
DCs	dendritic cells
DSCs	decidual stromal cells
ECL1–ECL3	three extracellular loops within G-protein-coupled receptor (GPCR)
ECM	extracellular matrix
EGF	epidermal growth factor
EGR1	early growth response 1 protein
ELA	Elabela,
ELA-32	Elabela-32, a mature, 32 amino acid containing, endogenous high affinity apelin receptor agonist
ELISA	enzyme-linked immunosorbent assay
eNOS	endothelial nitric oxide synthase
ER	endoplasmic reticulum
ERK1/2	extracellular signal-regulated kinase ½
FGR	fetal growth restriction (retardation)
FTDCs	first-trimester decidual cells
Gα, Gβ, Gγ	subunits of the heterotrimeric G proteins (G protein complex)
Gαi	activated Gα subunit of the G protein complex
GABA	gamma-aminobutyric acid
GABA-A receptor	gamma-aminobutyric acid receptor A
GABRP	gamma-aminobutyric acid receptor A π subunit
GEF	guanine nucleotide exchange factor
GDM	gestational diabetes mellitus
GDP	guanosine diphosphate
GH	growth hormone
GPCR	G-protein-coupled receptor
GRK	G-protein-coupled receptor kinase
GTP	guanosine triphosphate
hCG	human chorionic gonadotropin
HELLP syndrome	hemolysis, elevated liver enzymes and low platelets syndrome
HGF	hepatocyte growth factor, also known as scatter factor
HIF-1α	hypoxia-inducible factor 1 alpha
HLA-G	major histocompatibility complex (MHC) antigen, class I, G, also known as human leukocyte antigen G
HUVECs	human umbilical vein endothelial cells
ICL1–ICL3	three intracellular loops within G-protein-coupled receptor (GPCR)
IDO1	enzyme indoleamine 2,3-dioxygenase 1
IFN-γ	interferon gamma
IL-1β, IL-6, IL-11	interleukin-1β, -6, -11 respectively
ILT4 receptor	Ig-like transcript 4 inhibitory receptor
JAK	Janus kinase
LIF	leukemia inhibitory factor
Mφ	macrophage
M1	proinflammatory polarized macrophages
M2	anti-inflammatory polarized macrophages
*MAP2K1*	mitogen-activated protein kinase kinase 1 encoding gene
*MAP2K2*	mitogen-activated protein kinase kinase 2 encoding gene
MAPK	mitogen-activated protein kinase
MC	mast cells
MDSCs	myeloid-derived suppressor cells
MEK1	mitogen-activated protein kinase kinase 1
MEK2	mitogen-activated protein kinase kinase 2
MMP1, MMP-2, MMP12, MMP14, MMP15	matrix metalloproteinases-1, -2, -12, -14 and -15, respectively
MT-MMPs	membrane-type matrix metalloproteinases
mTOR	mammalian target of rapamycin
NF-κB	nuclear factor kappa-light-chain-enhancer of activated B cells
NK cell	natural killer cell
p38 MAPKs	p38 mitogen-activated protein kinases
p70S6 kinase	ribosomal protein S6 kinase beta-1, also known as S6K1
PBHSCs	peripheral blood-derived hematopoietic stem cells
PDK1	phosphoinositide dependent kinase 1
PE	preeclampsia
PI3K	phosphoinositide 3-kinase
PIP2	phosphatidylinositol 4,5-bisphosphate
PIP3	phosphatidylinositol (3,4,5)-trisphosphate
PKC	protein kinase C
PLC	phospholipase C
PRL	prolactin
RhoA	Ras homolog gene family member A
RIC8	resistance to inhibitors of cholinesterase 8 homolog A
ROS	reactive oxygen species
RTKs	receptor tyrosine kinases
S6K1	ribosomal protein S6 kinase beta-1, also known as p70S6 kinase
sCX3CL1	soluble form of chemokine CX3CL1 (fractalkine)
SNARE	vesicle-associated v-soluble N-ethylmaleimide-sensitive factor attachment protein receptor
SP	signal peptide
STAT	signal transducer and activator of transcription protein
STX13	protein syntaxin 13
TACE	tumor necrosis factor alpha-converting enzyme, also known as a disintegrin and metalloproteinase domain-containing protein 17 (ADAM17)
TLRs	toll-like receptors
TM1–TM7	seven hydrophobic α-helical transmembrane segments or domains within a G protein-coupled receptor (GPCR)
TNF-α	tumor necrosis factor alpha
VAMP3	vesicle-associated membrane protein 3
VCAM-1	vascular cell adhesion molecule 1 also known as vascular cell adhesion protein 1
VCTs	villous cytotrophoblasts
VEGF-A	vascular endothelial growth factor isoform A
VEGFR-2	vascular endothelial growth factor receptor type 2
V/EVTI	vascular/extravascular tissue index
